# A geminivirus betasatellite damages the structural and functional integrity of chloroplasts leading to symptom formation and inhibition of photosynthesis

**DOI:** 10.1093/jxb/erv299

**Published:** 2015-06-25

**Authors:** Dhriti Bhattacharyya, Prabu Gnanasekaran, Reddy Kishore Kumar, Nirbhay Kumar Kushwaha, Veerendra Kumar Sharma, Mohd Aslam Yusuf, Supriya Chakraborty

**Affiliations:** Molecular Virology Laboratory, School of Life Sciences, Jawaharlal Nehru University, New Delhi-110 067, India

**Keywords:** Betasatellite, βC1, chloroplast, geminivirus, photosynthesis, veinal chlorosis.

## Abstract

Geminivirus infection often causes severe vein clearing symptoms in hosts. Recently a betasatellite has emerged as a key regulator of symptom induction. To understand the host–betasatellite interactions in the process of symptom development, a systematic study was carried out involving symptoms induced by a betasatellite associated with radish leaf curl disease (RaLCB) in *Nicotiana benthamiana.* It has been found that βC1 protein localized to chloroplasts of host cells, and RaLCB lacking βC1, which failed to produce symptoms, had no effect on chloroplast ultrastructure. Vein flecking induced by transiently expressed βC1 was associated with chloroplast ultrastructure. In addition, the betasatellite down-regulates expression of genes involved in chlorophyll biosynthesis as well as genes involved in chloroplast development and plastid translocation. Interestingly, the expression of key host genes involved in chlorophyll degradation remains unaffected. Betasatellite infection drastically reduced the numbers of active reaction centres and the plastoquinol pool size in leaves exhibiting vein clearing symptoms. Betasatellite-mediated impediments at different stages of chloroplast functionality affect the photosynthetic efficiency of *N. benthamiana.* To the best of the authors’ knowledge, this is the first evidence of a chloroplast-targeting protein encoded by a DNA virus which induces vein clearing and structurally and functionally damages chloroplasts in plants.

## Highlight

A satellite DNA-encoded protein (βC1) is localized in the chloroplast. The intercellular events associated with βC1-induced photosynthetic inhibition and vein clearing symptom formation are discussed.

## Introduction

Viruses infect plants systemically and, like any other compatible host–pathogen system, use the host machinery to survive and multiply inside the cells. Subcellular organelles such as mitochondria, the endoplasmic reticulum (ER), and the nucleus are exploited by plant viruses for replication and transcription of their genomes (Rubino *et al.*, 2001; McCartney *et al.*, 2005).

Chloroplasts, the structural units of the photosynthetic machinery of green plants, are devoid of RNA silencing machinery, the major defensive mechanism of plants against viruses. Knowledge from different plant virus systems has indicated that chloroplasts are the prime target of RNA virus-mediated pathogenesis (Reinero and Beachy, 1989; Takahashi and Ehara, 1992). Direct or indirect interactions of RNA virus-encoded proteins with the chloroplastic proteins contribute significantly to viral pathogenesis (Reinero and Beachy, 1989; Xiang *et al.*, 2006; Kong *et al.*, 2014). With its unique origin as an endosymbiont converted into a subcellular organelle, the chloroplast is speculated to have the potential to harbour the tools necessary for replication and transcription of viruses (Wei *et al.*, 2010). Moreover, the chloroplast’s dual roles, as the home of photosynthesis and a hub of defence response (Jin *et al.*, 2007; Caplan *et al.*, 2008), make them attractive targets for viral pathogens as evidenced by the cases of *Tobacco mosaic virus* (TMV), *Plum poxvirus*, and *Potato virus Y*. Two of the nuclear-encoded chloroplast proteins, AtpC and RCA, are involved in the formation of the virus replication complex (VRC) in the cytoplasm (Bhat *et al.*, 2013). The coat protein of TMV localizes in the thylakoid membrane, binds to the photosystem II (PSII) complex, makes a scaffold mimicking the light-harvesting complex II (LHCII) complex, and functionally hampers the PSII machinery (Reinero and Beachy, 1989; Ma *et al.*, 2008). These reports outline a prognostic relationship between RNA viruses and the chloroplasts. However, to date, very little systematic study has been undertaken to understand the consequence of DNA virus infection on the chloroplast and the photosynthetic machinery.

Geminiviruses (family *Geminiviridae*) are plant-infecting DNA viruses which are considered one of the major threats for crop production worldwide. Geminiviruses possess circular single-stranded DNA genomes of ~2.7kb and are encapsidated in twinned quasi-isometric particles. These viruses are classified, depending on the insect vector and genomic characteristics, into seven genera: *Becurtovirus*, *Begomovirus*, *Curtovirus*, *Eragrovirus*, *Mastrevirus*, *Topocuvirus*, and *Turncurtovirus* (Adams *et al.*, 2013). Begomoviruses (type species: *Bean golden mosaic virus*) are transmitted by whiteflies (*Bemisia tabaci* Genn.) and possess either a monopartite (a single DNA A-like component) or a bipartite (consisting of two DNA components: DNA A and DNA B) genomic organization. The geminivirus–plant interface is populated by diverse host cellular and physiological pathways that are intrinsically manoeuvred by viruses for propagation and establishment of infection (Sahu *et al.*, 2014). A betasatellite, a subviral DNA molecule of 1.3kb, is often associated with a number of monopartite viral genomes (DNA A-like component) (Briddon and Markham, 2000; Saunders *et al.*, 2000, 2003). The betasatellite encodes a 13.5kDa protein named βC1, a suppressor of gene silencing, a symptom determinant, and capable of functionally replacing the DNA B-encoded movement protein (Cui *et al.*, 2005; Saeed *et al.*, 2007; Singh *et al.*, 2012). The βC1 protein interacts with *S*-adenosylhomocysteine hydrolase (SAHH) to suppress methylation-mediated transcriptional gene silencing (Yang *et al.*, 2011). The βC1 protein interferes with the plant’s AS1–AS2 protein-dependent developmental pathway (Yang *et al.*, 2008), becomes phosphorylated by host SnRK1, determining pathogenicity (Shen *et al.*, 2011), and, in order to facilitate vector evasion, suppresses the host’s terpene biosynthesis pathway by interacting with the MYC 2 transcription factor (Li *et al.*, 2014). The promoter activity of the betasatellite, a phloem-specific but not exclusively phloem-limited phenomenon (Ding *et al.*, 2009; Zhang *et al.*, 2012), has been reported to be important for symptom development.

The earliest mention of plants with yellow leaves appeared in a 1300-year-old Japanese poem penned by Empress Koken (Saunders *et al.*, 2003). Later scientific studies confirmed that a geminivirus and its associated betasatellite could reproduce the leaf yellowing symptom (Saunders *et al.*, 2003), and association of the betasatellite with monopartite begomoviruses such as *Ageratum yellow vein virus*, *Bhendi yellow vein mosaic virus*, *Cotton leaf curl Multan virus*, *Eupatorium yellow vein virus*, *Tomato yellow leaf curl China virus*, and *Radish leaf curl virus* has been found to be essential for the induction of typical disease symptoms (Supplementary Fig. S1 available at *JXB* online; Jose and Usha, 2003; Mansoor *et al.*, 2003; Saunders *et al.*, 2003). Although betasatellite infection is known to be associated with yellow vein diseases, the physiological and intercellular response associated with betasatellite-mediated veinal chlorosis has not been explored yet.

In this study, the effect of the betasatellite which is associated with radish leaf curl disease (RaLCB referred to as β in the text) on the physiology of the model plant *Nicotiana benthamiana* was studied and the basis of veinal chlorosis symptom formation was investigated. It was found that while *Tomato leaf curl New Delhi virus* DNA A (ToLCNDV; referred to as A in the text) infection does not damage the chloroplast structure of the host cell, co-infection of A and β severely alters the chloroplast ultrastructure. The results demonstrate that protein βC1 is responsible for veinal chlorosis formation and is capable of localizing in the host cell chloroplast. This is the first report so far of a DNA virus-encoded protein causing structural and functional damage to this indispensable organelle, leading to symptom formation.

## Materials and methods

### Plant materials, virus strains, and virus inoculations

*Nicotiana benthamina* plants were grown in a glass house at 25 °C and 70% relative humidity in 16h of light and 8h of dark. Infectious tandem repeat constructs of ToLCNDV (referred to as A; GenBank accession no. U15015; Chakraborty *et al.*, 2008) and RaLCB (referred to as β; GenBank accession no. EF175734; Singh *et al.*, 2012) were the property of the authors. For the RaLCB∆βC1 infectious clone, primers were designed to amplify the RaLCB genome excluding the βC1 open reading frame (ORF). A partial tandem repeat of 1.0kb of the RaLCB∆βC1 genome was cloned in pCAMBIA2300. *Agrobacterium tumefaciens*-mediated inoculation was performed according to Singh *et al.* (2012).

For the transient expression assay, βC1 from RaLCB was amplified with primers containing *Nco*I and *Not*I sites at the forward and reverse primer, respectively, and cloned downstream of the phloem-specific promoter of AtSUC2 in the pEPS1 vector (Stadler *et al.*, 2005). Fully grown leaves of 3-week-old *N. benthamiana* plants were infiltrated with *A. tumefaciens* cells containing the pEPS1-βC1 construct.

### Chlorophyll estimation and transmission electron microscopy

To measure the chlorophyll content, fresh uppermost leaves from three biological and three technical replicates (nine replicates in total) of mock-inoculated and symptomatic leaves of virus-infected plants were collected, and absorption at 645, 663, and 652nm was measured following homogenization in 80% acetone. The amount of chlorophyll was calculated according to Arnon’s equation. The grand means of values were subjected to analysis of variance (ANOVA) followed by Scheffe test at the 0.05 significance level.

For transmission electron microscopy (TEM), samples were fixed in a solution of 2.5% glutaraldehyde overnight followed by addition of 0.1M sodium-phosphate buffer (pH 7.0) and treatment with the secondary fixative 1% OsO_4_. Tissues, dehydrated with acetone, were embedded in araldite and were subjected to ultrathin sectioning followed by staining with uranyl acetate. Samples were examined with a JEOL 2100F electron microscope. The images of replicate samples were analysed with ImageJ software for measuring the number and area of the chloroplasts (http://rsb.info.nih.gov/ij).

### Measurement of chlorophyll *a* fluorescence transients and photosynthetic gas exchange

Both mock- and virus-inoculated plants were dark adapted for 2h and chlorophyll *a* fluorescence was measured in intact attached leaves. Three plants for each group (i.e. mock, A, and A+β inoculated) were used and six readings for each leaf were recorded. The polyphasic chlorophyll *a* fluorescence transient (10 μs to 1 s) was measured using the Handy-PEA fluorimeter (Hansatech, Kings Lynn, UK) and the OJIP transients were analysed using the JIP test (Strasser *et al.*, 2000). Red light (peak at 650nm) of intensity 3000 μmol m^–2^ s^–1^, provided by an array of three high-intensity light-emitting diodes, was used to induce the transient. The data were obtained at every 10 μs (from 10 μs to 0.3ms), every 0.1ms (from 0.3ms to 3ms), every 1ms (from 3ms to 30ms), every 10ms (from 30ms to 300ms), and every 100ms (from 300ms to 1s).

From the JIP test, different parameters providing structural and functional information regarding the photosynthetic apparatus and energy fluxes were calculated by Biolyzer v.3.0.6 software (Strasser *et al.*, 2000).

Snapshot measurement and analysis of photosynthetic carbon assimilation (Photo), stomatal conductance (Cond), and the intercellular CO_2_ concentration (Ci) of biological replicates of mock-, A-, and A+β-inoculated plants, as well as pEPS1- and pEPS1-βC1-infiltrated plants, were performed by the portable LI-6400XT photosynthesis measurements system (LI-COR, Lincoln, NE, USA).

### Protein extraction and two-dimensional gel electrophoresis followed by MALDI-TOF, LC-MS-MS

Fresh tissue collected from either mock-infected plants or symptomatic leaves of A- and A+β-infected plants were frozen in liquid N_2_ and homogenized in buffer containing 50mM TRIS-HCl (pH 7.6), 100mM NaCl, 1% Triton-X, 5mM β-mercaptoethanol, 1mM EDTA, and protease inhibitor cocktail (Sigma, St Louis, MO, USA). The homogenates were centrifuged at 14 000rpm for 15min and the protein concentration of the supernatant was assayed by the Bradford method. Proteins were concentrated with a 2D Clean-Up Kit (GE Healthcare, NJ, USA) and were dissolved in 50 μl of sample buffer containing 7M urea, 2M thiourea, 5% CHAPS, 2% 2-mercaptoethanol, and 5% ampholines pH 3.5–10. A 500mg aliquot of proteins, quantified with a 2D Quant Kit (GE Healthcare), were used to rehydrate the 13cm IPG strips of pH 3–10. Isoelectric focusing was performed on an IPGphor unit (GE Healthcare).

Before the second dimensional electrophoresis, strips were reduced with dithiothreoitol (DTT) and alkylated with iodacetamide. The second dimension was run in 12% polyacrylamide gels. Quantitative analysis of the three biological replicated gels was executed both manually and using the Image Master Platinum v.6.0 software. Differentially expressed protein spots were excised manually and were processed further for sequencing through matrix-associated laser desorption ionization time-of-flight mass spectrometry (MALDI-TOF MS) (ABI Sciex 5800 TOF/TOF system, USA).

### Total RNA extraction and quantitative reverse transcription–PCR analysis

Total RNAs from leaf tissues of A- and A+β-infected plants were extracted using TRI Reagent (Sigma-Aldrich) and treated with DNase I. First-strand DNA synthesis was carried out using oligo(dT) primer and MuMLV reverse transcriptase (Fermentas, USA). For all the genes, primers had been designed according to the sequences already published for either *N. benthamiana* or *Nicotiana tabacum* or obtained from the NCBI genome database (Supplementary Table S6 at *JXB* online). The primers for the chloroplastic CSS1 precursor gene were designed using the sequence available for *Pisum sativum*. The measured target mRNA amounts were normalized against the corresponding actin mRNA amplified from the same total RNA samples and obtained from the same reverse transcription reactions in parallel PCR runs.

Grand means of RT–PCR data were calculated from three replicates of A- and A+β-infected samples, and the values obtained were subjected to sample *t*-test to determine the significant differences between A- and A+β-infected samples at the 0.05 significance level.

### Subcellular localization of βC1 by confocal fluorescence microscopy

To ascertain the subcellular localization of βC1 inside the host cell, the ORF was cloned in the pCXDG vector (obtained from the Arabidopsis stock center; http://www.arabidopsis.org/) as a 5′ in-frame fusion with green fluorescent protein (GFP) and the protein was transiently expressed in *N. benthamiana* via agroinfiltration. The βC1 ORF of RaLCB was amplified with specific primers (5′-GAAGCTTATGACGATCAAATACAAAAACC-3′ and 5′-GTCGACTTATACAGATGAACGCGTATACAC-3′) and cloned into the *Xcm*I-digested pCXDG vector to produce pCXDG-βC1. *Agrobacterium tumefaciens* strain GV3101 was transformed either with pCXDG or with pCXDG-βC1. Leaves of 3-week-old *N. benthamiana* plants were infiltrated with the *Agrobacterium* cultures. The epidermal cells of the infiltrated leaves were visualized under an Andor Spinning Disk Confocal Microscope (Andor, Belfast, Ireland) at 72h post-infiltration. For detection of 4′,6-diamidino-2-phenylindole (DAPI) fluorescence, a 340–380nm excitation filter and a 435–485nm emission filter were used. For GFP fluorescence, a 465–495nm excitation filter and a 515–555nm emission filter were used. For chlorophyll autofluorescence, a 543nm excitation filter and a 650nm emission filter were used. For merging and analysis of the captured imags, Andor iQ2.7 software was used.

### Detection of βC1 protein in chloroplasts of infected *N. benthamiana* by immunoelectron microscopy

The pCXSN-HA-βC1 construct was made by amplifying the βC1 ORF using specific primers (5′-GAAGCTTATG ACGATCAAATACAAAAACC-3′ and 5′-GTCGACTTATACA GATGAACGCGTATACAC-3′) and subsequently cloned in the *Xcm*I-digested pCXSN-HA vector (http://www.arabidopsis.org/). N-terminal double HA-tagged βC1 was amplified from the pCXSN-HA-βC1 construct using a forward primer containing the HA-tag sequence (5′-CCATGGATGTACCCATACGATGT TCCAGATTACGCTCCAATACTGAAGCTTATG-3′) and the reverse primer (5′-GCGGCCGCTTATACAGATGAACGCGTA TACAC-3′). The βC1 ORF located within the monomeric clone of RaLCB was replaced with an N-terminal double HA-tagged βC1 ORF. A partial tandem repeat of this chimeric clone (βHAβC1) was generated following Singh *et al.* (2012), and agroinoculation of *N. benthamiana* plants was carried out with either A or A+βHAβC1. At 28 days post-inoculation (dpi), fixation of the uppermost leaves of *N. benthamiana* inoculated with either A or A+βHAβC1 was carried out as described by Cui *et al.* (2005) with slight modification. The ultrathin sections were cut and mounted on nickel grids. After blocking, the grids were incubated in anti-HA antibody (Sigma) for 1h. The grids were washed with phosphate-buffered saline (PBS) several times and incubated in anti-mouse IgG–gold antibody produced in goat. After washing several times the grids were subsequently visualized under a transmission electron microscope (JEOL 2100F).

## Results

### Betasatellite causes degradation of chlorophyll in infected leaves of *N. benthamiana*

**Fig. 1.**
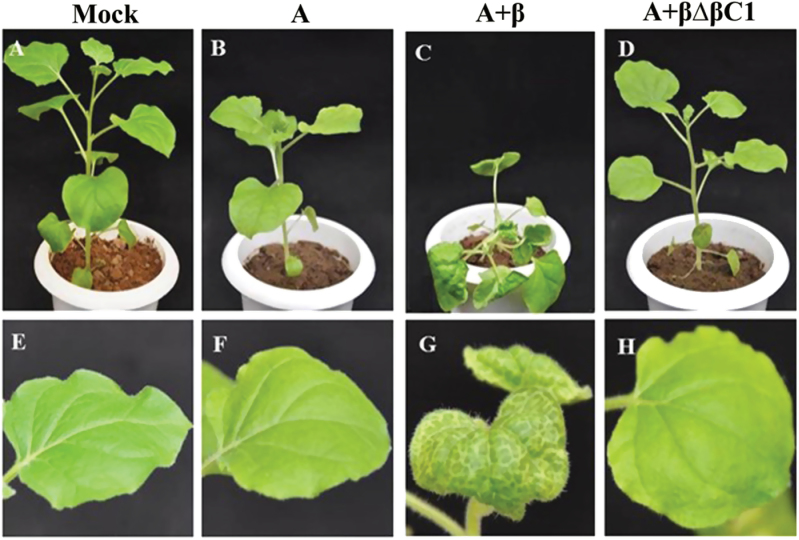
Veinal chlorosis induced by protein βC1 of RaLCB in *N. benthamiana*. (A–D) The plant phenotype following inoculation with the respective combinations as indicated above each photograph at 28 dpi. (E–H) Close-up view of symptomatic upper leaves of the same plants.

**Fig. 2.**
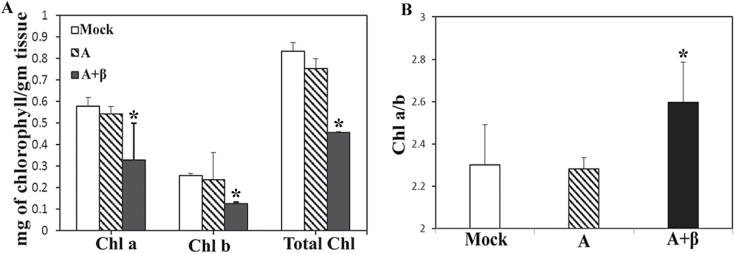
RaLCB infection causes degradation of chlorophyll in *N. benthamiana.* (A) Relative amount of chlorophyll *a* (mg g^–1^), chlorophyll *b* (mg g^–1^), and total chlorophyll (mg g^–1^) and (B) the chlorophyll *a*/*b* ratio from leaves of mock- (control), A-, and A+β-inoculated plants at 28 dpi. Each bar represents a grand mean ±SD of three experimental means. The chlorophyll concentration determinations were carried out for three biological and three technical replicates (a total of nine replicates) and the grand means of values for chlorophyll *a*, chlorophyll *b*, total chlorophyll and the chlorophyll *a*/*b* ratio were calculated and subjected to analysis of variance followed by post-hoc Scheffe test to determine significant differences between mock-, A-, and A+β-infected samples at the 0.05 significance level. Bars marked with an asterisk (*) represent samples showing a significant difference in level.

The betasatellite involved in leaf curl disease of radish (RaLCB) interacts with ToLCNDV DNA A and substitutes its cognate DNA B (Singh *et al.*, 2012). When co-inoculated with A in the model plant *N. benthamiana*, β initiated leaf curling and vein clearing at 14 dpi on minor veins which later on turned to severe veinal chlorosis affecting the mid-veins and leaf lamina at 28 dpi (Fig. 1). Samples from leaves of the same physiological age of mock-inoculated, A-inoculated, and A+β-inoculated plants at 28 dpi were collected and their chlorophyll *a*, chlorophyll *b*, and total chlorophyll contents, and chlorophyll *a*/*b* ratio were measured. It was found that betasatellite infection caused a 43.3% and 51% decrease in chlorophyll *a* and *b* content, respectively. These changes were manifested in a 45.2% decrease in total chlorophyll and a 14.9% increase in the chlorophyll *a*/*b* ratio of the non-infected control plants, whereas in the case of A-inoculated plants, no significant difference was observed as compared with mock-inoculated plants (Fig. 2). Since the effect of A on chlorophyll content is minimal, further study was concentrated on analysing the comparative change in the physiological condition of A- and A+β-infected plants.

**Fig. 3.**
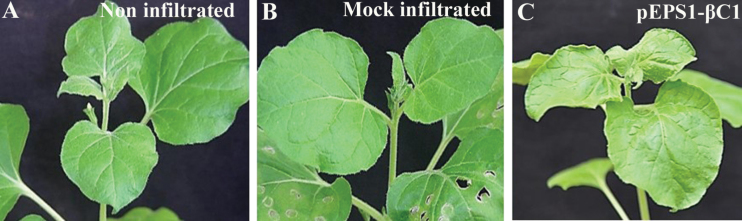
Transiently expressed βC1-induced symptom formation in systemic leaves of *N. benthamiana* at 7 dpi. (This figure is available in colour at *JXB* online.)

RaLCB lacking the βC1 gene failed to induce symptoms in plants. This result suggested a role for βC1 in vein clearing (Fig. 1). However, accumulation of betasatellite and helper virus in these plants could be detected. Transient assay of βC1 under the influence of the AtSuc2 promoter in *N. benthamiana* showed that at 7 dpi veins of the upper systemic leaves from infiltrated plants exhibited deformities such as mild crumpling of leaves along with vein flecking/yellowing (Fig. 3C). The total chlorophyll level in these symptomatic leaves was reduced by 40% (Supplementary Table S1 at *JXB* online). The observed reduction in chlorophyll level following betasatellite infection led to further determination of the impact of the betasatellite on the chloroplast machinery of plants.

### Betasatellite infection causes ultrastructural damage of chloroplasts

**Fig. 4.**
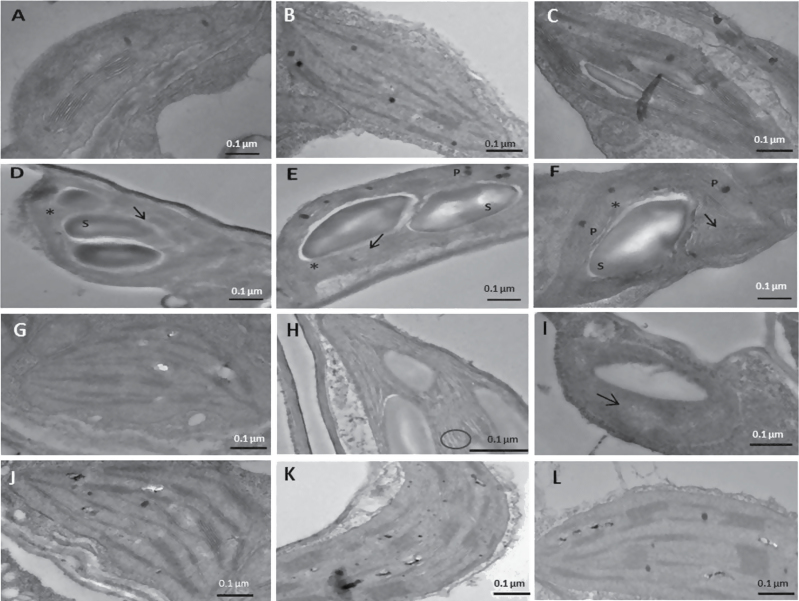
Chloroplast structure and thylakoid organization in *N. benthamiana* plants at 28 dpi as observed by transmission electron microscopy. (A) A normal chloroplast in a leaf from a mock-inoculated plant. (B and C) Chloroplasts from a leaf of an A-inoculated plant do not show alteration in plastid structure. Chloroplasts from a leaf of an A+β-infected plant (D–F) lack organized grana with the partial disappearance of thylakoids and are irregularly shaped. Agroinfiltrated *N. benthamiana* plants transiently expressing βC1 also show differential appearance of chloroplasts at 8 dpi. (G) Chloroplasts from a leaf of a pEPS1 vector-infiltrated plant. (H and I) Chloroplasts from leaves of plants infiltrated with pEPS1-βC1 show enhanced starch accumulation and swollen thylakoids with increased space between grana stacks, and almost complete disappearance of thylakoids. Chloroplasts from (K and L) the leaves of A+RaLCB∆βC1 show no difference from the chloroplasts from (J) the leaf of an A-inoculated plant. P, plastoglobules; S, starch accumulation; an asterisk (*) indicates an area of thylakoid which has disappeared; an arrow indicates lack of organized grana; an open circle indicates a swollen thylakoid.

To investigate the effect of betasatellite infection on the physical organization of the chloroplast, the ultrastructure of this organelle was studied by TEM. No significant alteration in the chloroplast structure of A-inoculated plants in comparison with the mock-treated plants was seen. However, images of chloroplasts from A+β-infected plants, when compared with mock- and A-inoculated plants of the same physiological age, showed partial disappearance of the thylakoid structure and the stacking of grana was severely affected (Fig. 4A–F). There was a large accumulation of plastoglobulin and starch inside the chloroplasts of the betasatellite-infected plants (Fig. 4D–F) and the majority of these chloroplasts were irregularly shaped as compared with the mock- and A-inoculated plants (Fig. 4A–C). However, chloroplasts from RaLCB∆βC1-infected plants (Fig. 4K–L) showed no difference in organization of thylakoids and grana stacking from chloroplast from A-infected plants (Fig. 4J). This observation again suggested that βC1 mediated the damage to the chloroplast ultrastructure.

**Fig. 5.**
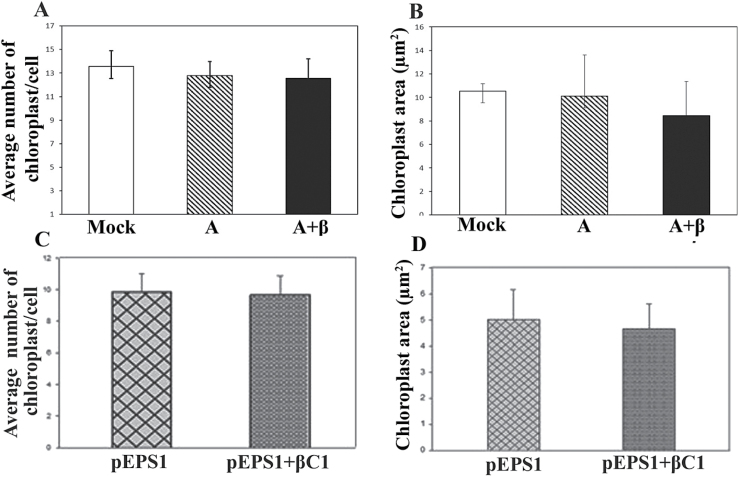
Effect of RaLCB infection on chloroplast number and area. Comparison of chloroplast area in symptomatic leaves of (A) mock-, A-, and A+β-inoculated plants at 28 dpi. (C) pEPS1 vector- and pEPS1-βC1-infiltrated plant at 8 dpi. Comparison of number of chloroplasts in symptomatic leaves of (B) mock-, A-, and A+β-inoculated plants at 28 dpi (D) pEPS1 vector- and pEPS1-βC1-infiltrated plant at 8 dpi. Bars represent means ±standard errors for at least 20 replicates per treatment. Grand means of data were calculated from replicates of infected samples and the means obtained were subjected to independent sample *t*-test to determine significant differences between samples at the 0.05 significance level.

The ultrastructure of chloroplasts from leaves of pEPS1-βC1-infiltrated plants (Fig. 4H, I) was compared with that of those of pEPS1 vector-infiltrated plants (Fig. 4G). The pEPS1-βC1-infiltrated leaf samples, quite similarly to A+β-infected plants, showed enhanced starch accumulation and swollen thylakoids with increased space between grana stacks and the almost total disappearance of thylakoids, confirming the role of βC1 in inducing damage in chloroplasts. No significant change in the average number and size of the chloroplasts in β-infected plants with respect to mock- and A-infected plants was found (Fig. 5).

### *β*C1 expression severely reduces photosynthetic efficiency of the host

**Fig. 6.**
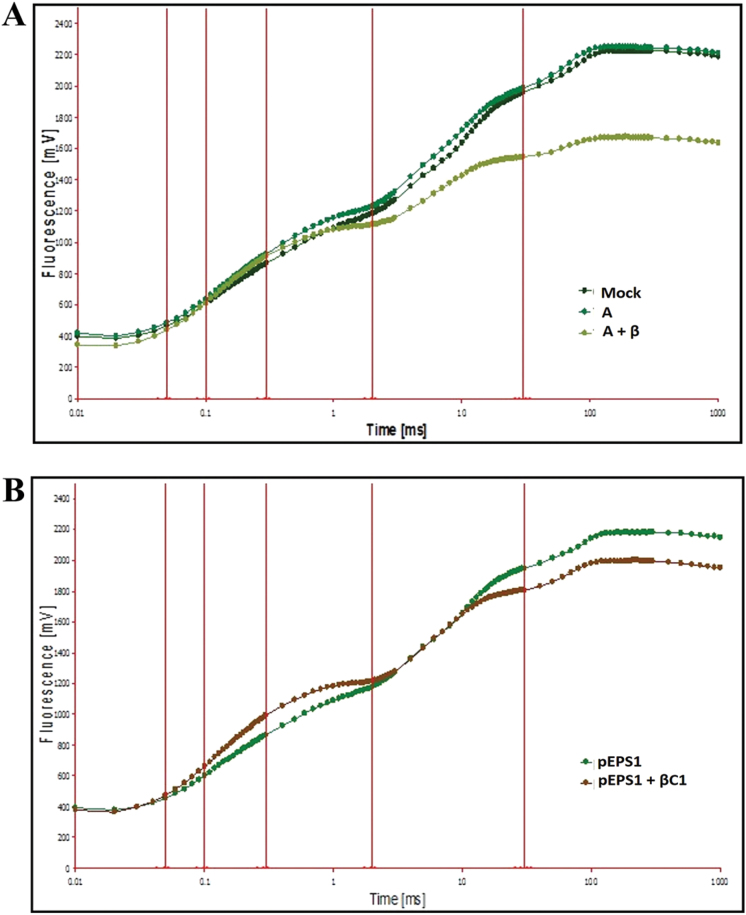
Chlorophyll *a* fluorescence induction curve of infected symptomatic leaves from *N. benthamiana*. Chlorophyll *a* fluorescence induction curve of dark-adapted plants infected with (A) mock, A, and A+β at 28 dpi and of plants agroinfiltrated with (B) pEPS1 and pEPS1-βC1 at 7 dpi. The arrowhead indicates the formation of a K peak in the OJIP curve. (This figure is available in colour at *JXB* online.)

Chlorophyll *a* fluorescence is regarded as the ‘signature of photosynthesis’, and detailed analysis of the photosynthetic energy fluxes is possible by using polyphasic kinetics of the fast chlorophyll *a* fluorescence transient as a tool. Chlorophyll fluorescence transients revealed that the quantum yield of photochemistry of PSII was reduced in plants inoculated with A+β compared with those inoculated with A and control plants (Fig. 6A). The same pattern of deviation was observed in the appearance of the curve for pEPS1-βC1-infiltrated plants in comparison with pEPS1-infiltrated plants (Fig. 6B). The area above the OJIP curve (i.e. the pool size of the electron acceptor Qa on the reducing side of PSII) decreased down to almost half of that for control and A-inoculated plants, indicating that betasatellite infection blocked the electron transport from Qa^–^ to intersystem electron acceptors. Formation of a pronounced K peak in pEPS1-βC1-infiltrated plants indicated that damage occurred in the oxygen-evolving complex (OEC).

**Fig. 7.**
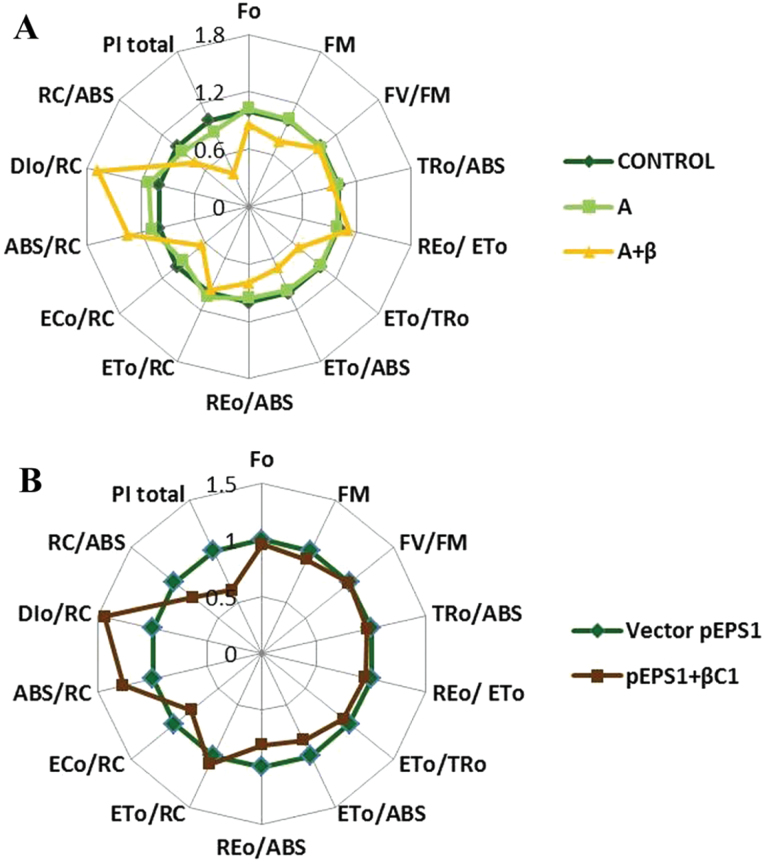
Influence of betasatellite infection on selected functional and structural JIP test parameters. (A) Changes in parameters in A- and A+β-infected symptomatic leaves from *N. benthamiana* at 28 dpi (radar plot centre=0.0, maximum=1.8) relative to mock-infected plants. (B) Changes in parameters in symptomatic leaves from *N. benthamiana* transiently expressing pEPS1-βC1 at 7 dpi (radar plot centre=0.0, maximum=1.5) relative to plants infiltrated with pEPS1 vector. (This figure is available in colour at *JXB* online.)

In OJIP analysis, the O–I phase spans from the phase of trapping of excitons by PSII up to plastoquinone (PQ) reduction, and the I–P phase describes the electron transfer starting at plastoquinol (PQH_2_) to the end acceptors on the PSI acceptor side. Different biophysical parameters (Supplementary Table S2 at *JXB* online) related to the PSII behaviour pattern extracted through OJIP analysis were plotted in a spider plot (Fig. 7A, B). Most of these parameters were affected by betasatellite infection contributing to the overall decreased photosynthetic activity of the leaf, while A infection caused minimal change in these parameters. The total electron carriers per reaction centre (EC_0_/RC) were decreased by 35% upon betasatellite infection. Parameters for the quantum yields (i.e. TR_0_/ABS, ET_0_/ABS, and RE_0_/ABS) were decreased. The overall performance index (PI) of photosynthesis carries the cumulative effects of all the energy fluxes involved in the successive steps. The PI_total_ of the A+β-infected symptomatic leaves was severely compromised (~40% compared with the mock treatment) though in A-infected leaves this decrease was 12% (Supplementary Fig. S2). ABS/RC (i.e. total absorption per active RC) showed a 35% increase in β-infected plants.

**Fig. 8.**
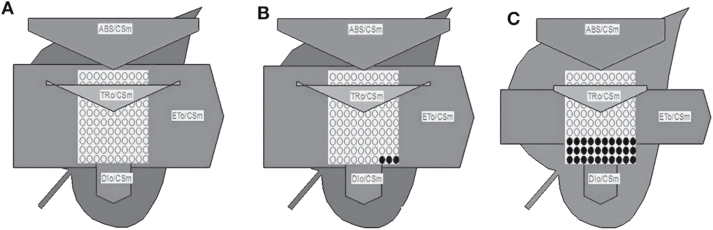
Effect of betasatellite infection on energy fluxes involved in photosynthesis. Leaf pipeline model showing the proportion of phenomenological energy fluxes per excited cross-sectional area on the uppermost leaf of plants inoculated with (A) mock, (B) A and (C) A+β. Each open circle indicates an individual active reaction centre, and a filled circle indicates a damaged/inactivated reaction centre. ABS/CS, absorption flux per CS; TR/CS, trapped energy flux per CS; ET/CS, electron transport flux per CS; DI/CS, dissipated energy flux per CS.

The phenomenological leaf pipeline model revealed that electron transport per excited cross-section (ET_0_/CS_m_) was decreased because of the inactivation of the RC complex (Fig. 8). This inactivation decreases the density of active (Q_a_ to Q_a_^–^ reducing) RCs. The flux for the energy dissipation per excited cross-section (DI_0_/CS_m_) was increased. The amount of this increase was 67% as indicated in the spider plot. The pipeline model also showed that the photon flux absorbed per excited cross-section (ABS/CS) was reduced due to betasatellite infection.

The PI of the symptomatic leaves of pEPS1-βC1-infiltrated plants was decreased by 30% compared with that of the empty vector-infiltrated plants. The other structural and functional parameters depicting different energy fluxes involved in the photosynthetic energy cascades were also affected, following the reduction pattern as observed in the betasatellite-infected plants (Fig. 7A, B; Supplementary Fig. S2 at *JXB* online).

Snapshot measurement and analysis of photosynthetic carbon assimilation (Pn) and stomatal conductance (Cond) were performed. It was found that with respect to mock-treated plants, the photosynthetic efficiency of A+β-infected and pEPS1-βC1-infiltrated plants was reduced by 60% and 30%, respectively (Supplementary Table S3 at *JXB* online). Taken together, these results confirmed that mere transient expression of the protein βC1 indeed was capable of affecting the functional integrity of chloroplasts similar to RaLCB infection.

### *β*C1 protein localizes in the nucleus as well as chloroplasts

**Fig. 9.**
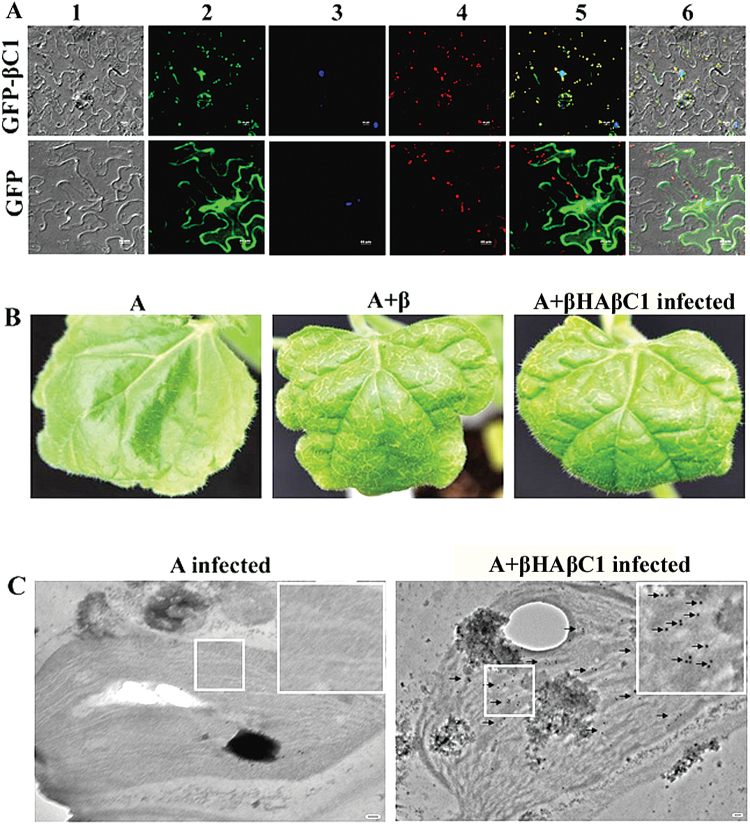
Subcellular localization of the βC1 protein. (A) *N. benthamiana* leaves were agroinfiltrated either with pCXDG-βC1 (upper panel) or with pCXDG (lower panel). The epidermal cells were visualized under confocal microscopy at 72h post-infiltration. Lanes 1–4 show the DIC images of *N. benthamiana* epidermal cells, localization of free GFP–βC1 or GFP, a nucleus stained with DAPI, and red autofluorescence of chloroplast, respectively. Lanes 5 and 6 represent the merged image without and with the DIC image, respectively. THe scale bar represents 20 μm. (B) N-terminal double HA-tagged βC1 induces veinal chlorosis and leaf curling similar to βC1 on *N. benthamiana*. (C) Detection of HAβC1 fusion protein in chloroplasts of an A+βHAβC1-infected plant by immunoelectron microscopy compared with an A-infected plant. The scale bar represents 100nm. The presence of the βC1 protein is indicated with a solid black arrow. The inset in (C) shows an enlargement of a chloroplast.

The presence of a nuclear localization signal (NLS) in the βC1 protein of the TYLCCNVY10 isolate has been reported to be essential for its localization to the nucleus (Cui *et al.*, 2005). The non-sequential search using the pseudo amino acid composition predicted RaLCB βC1 to be a chloroplast-localized protein (servers: Cell-PLoc, BaCelLo, and Euk-mPLoc 2.0). Confocal microscopy revealed that GFP–βC1 fluorescence merged with chlorophyll autofluorescence as well as with DAPI fluorescence, while the fluorescence from free GFP was distributed throughout the whole cell (Fig. 9A). This result showed that βC1 of RaLCB localized in the nucleus as well as the chloroplast, even in the absence of a conventional chloroplast transit peptide.

In addition, subcellular localization of βC1 in *N. benthamiana* mesophyll cells was determined out using pBIC-GFP-βC1 via agroinfiltration. The results indicate that GFP–βC1 fluorescence is merged with chlorophyll autofluorescence while fluorescence from free GFP was distributed throughout the whole cell (Supplementary Fig. S3 at *JXB* online).

To confirm further the localization of βC1 in chloroplasts during viral pathogenesis and to rule out the influence of the GFP tag on its localization, the presence of βC1 in chloroplasts of infected leaves was checked. *Nicotiana benthamiana* plants were inoculated with viral constructs (A, A+β, or A+βHAβC1). Notably, A+β- and A+βHAβC1-inoculated plants developed similar symptoms such as veinal chlorosis, downward curling, etc. (Fig. 9B), suggesting that βHAβC1 can produce the active N-terminal double HA-tagged βC1 protein for symptom development.

To corroborate the localization of βC1 in the chloroplasts of infected plants, immunoelectron microscopy was carried out. Detection of gold particles in the chloroplast from leaves of A+βHAβC1-, and not A alone-inoculated plants (Fig. 9C) further confirmed the chloroplast localization of βC1 during pathogenesis.

### Betasatellite infection alters the host cell proteome including expression of a number of chloroplastic proteins

**Fig. 10.**
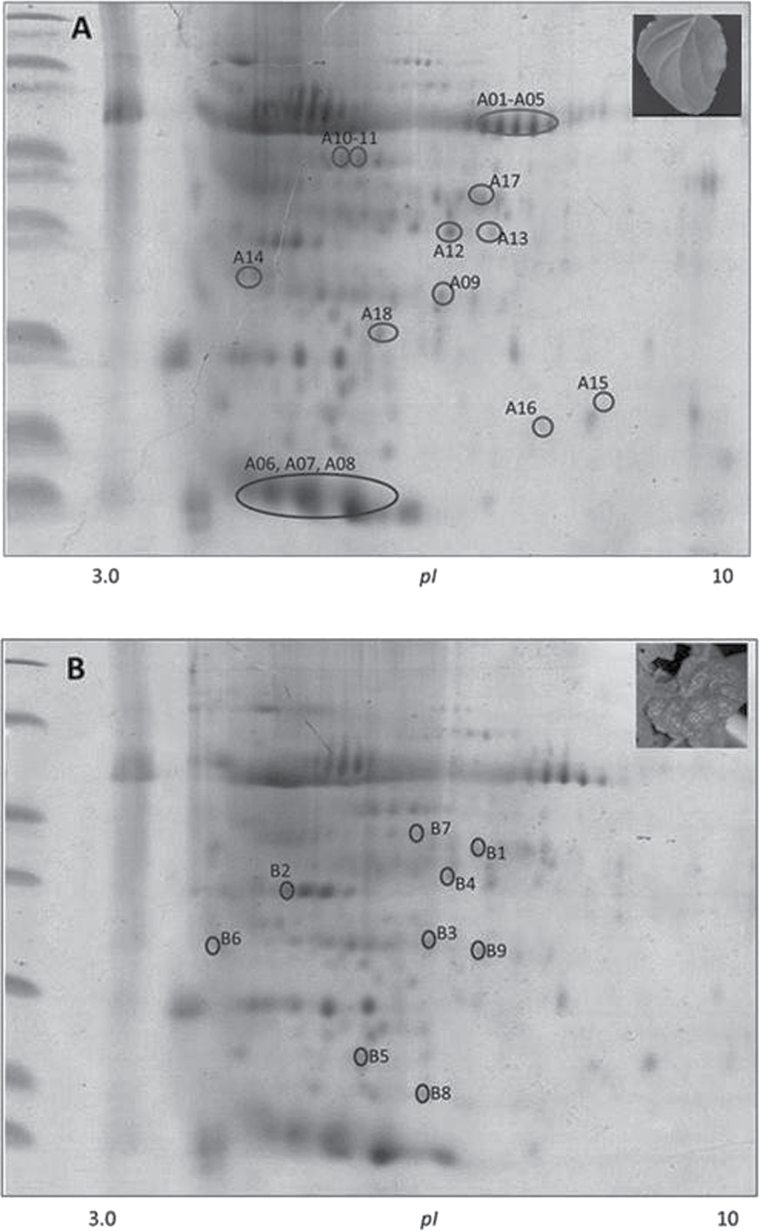
Changes in the proteome profile of *N. benthamiana* leaves after betasatellite infection. Two-dimensional gel electrophoresis of total protein extracted from plant leaves infected with (A) A and (B) A+β. The circles indicate the positions of proteins whose expression levels were altered by betasatellite infection. The spots on gel A and gel B were down-regulated and up-regulated, respectively. Changes in protein spots were calculated with ImageMaster 2D *Platinum* v6.0 software.

To identify the cellular proteins that were being differentially expressed following betasatellite infection, proteomic analysis by two-dimensional electrophoresis was performed with total protein from symptomatic leaves at 28 dpi from A- and A+β-inoculated *N. benthamiana* plants. A total of 27 protein spots were found to be consistently differentially expressed between the two protein samples (Fig. 10). The spots were excised manually and were analysed by MALDI-TOF/TOF. The spots were categorized in two classes; 18 spots that were down-regulated in A+β-infected leaves in comparison with A-infected plants were designated as A01–A18 (Supplementary Table S4 at *JXB* online) and nine spots that were up-regulated in A+β-infected leaves were designated as B01–B09 (Supplementary Table S5). The proteins which were down-regulated in the leaf due to betasatellite infection were found to be chloroplast targeted.

### Expression of plastid-targeted and photosynthetic genes was significantly altered by betasatellite infection

Virus infection might disturb the equilibrium between chlorophyll biosynthesis and degradation, leading to chlorotic symptom formation. Betasatellite infection results in reduction in the amount of chlorophyll and quantitative alteration in the level of chloroplast proteins. Therefore, the photosynthetic inhibition may occur due to a reduced level of chlorophyll and/or blockage at any point of the photosynthetic energy cascade by inhibition of the downstream enzyme(s). Hence, a study was carried out to investigate whether betasatellite infection alters the accumulation of mRNAs of the key enzymes involved in chlorophyll biosynthesis and degradation.

Each target mRNA was quantified by qRT–PCR using actin mRNA as the internal standard for relative RNA quantification (Supplementary Table S6 at *JXB* online). The mRNA isolated from symptomatic leaves from A-inoculated plants (28 dpi) were considered as the control to avoid the background of A-induced change in host mRNA.

**Fig. 11.**
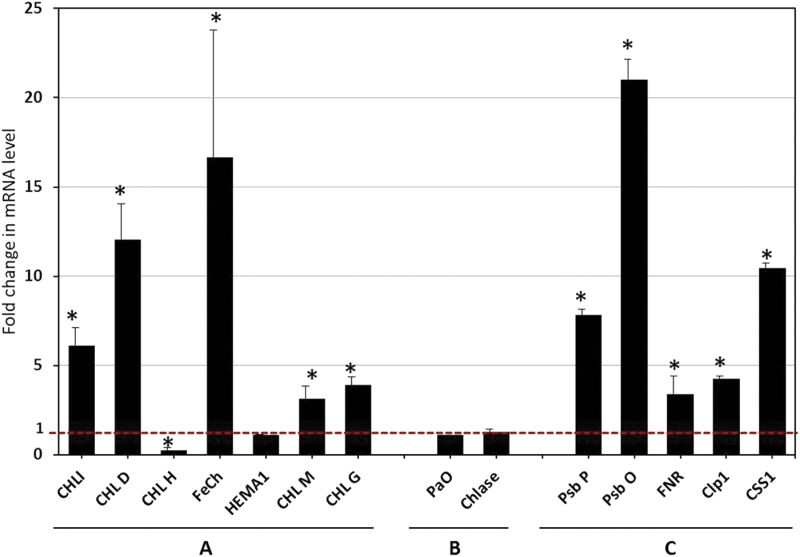
Effect of betasatellite infection on relative mRNA level of genes involved in chloroplast structure and function. *N. benthamiana* plants were agroinoculated with A and A+β. Quantitative RT–PCR was used to determine the relative mRNA level of target genes involved in (A) chlorophyll biosynthesis, (B) chlorophyll degradation, and (C) the structure and function of chloroplasts. The expression of the mRNAs was normalized with β-actin expression. Fold change of the transcript level in leaves infected with A+β in comparison with only A-infected leaves was calculated. Bars represent means ±standard errors for fold change obtained from at least three replicates per treatment. Grand means obtained were subjected to independent sample *t*-test to determine significant differences between A- and A+β-infected samples at the 0.05 significance level. Abbreviations of transcripts are given in the text.

In the chlorophyll biosynthesis pathway (Supplementary Fig. S4 at *JXB* online), changes in the mRNA level were measured for one enzyme at the beginning of the pathway, HEMA1-encoded Glu-tRNAreductase (GluTR; reducing Glu-tRNAGlu to glutamate-1-semialdehyde); two enzymes at the branch point, ferrochelatase (FeChe; involved in biosynthesis of haem) and Mg-chelatase (MgChe; consisting of three subunits ChlI, ChlD, and ChlH and involved in biosynthesis of chlorophyll); and two enzymes at the later stage of biosynthesis, namely Mg-protoporphyrin IX methyltranserase (ChlM) and chlorophyll synthase (ChlG) (Wettstein *et al.*, 1995). The enzymes involved in the chlorophyll biosynthesis pathway, except GluTR and ChlH, were significantly down-regulated (3- to 16-fold) in betasatellite-infected leaves (Fig. 11A). GluTR showed a statistically non-significant decrease in the transcript level, while subunit H of Mg-chelatase showed increased accumulation of mRNA in betasatellite-infected leaves.

Two enzymes involved in the chlorophyll degradation pathway were investigated to understand the nature of the equilibrium between the biosynthesis and degradation pathway (Supplementary Fig. S4 at *JXB* online). Notably, the transcript levels of both chlorophyllase, the first enzyme in chlorophyll breakdown pathway, and pheophorbide A oxygenase 1 (PaO), the first enzyme in the ring opening step responsible for converting pheide a to the first colourless product pFCC, were marginally reduced (Fig. 11B) and statistical analysis showed no significant difference between the transcripts in A- and A+β-infected plants at the 0.05 significance level.

Since the chloroplasts of betasatellite-infected cells were found to be convoluted in structure with disturbed organization of grana, and as the comparative proteome profile indicated that betasatellite induced down-regulation of proteins involved in normal development of the chloroplast, transcriptional levels of chloroplastic ClpP1 and DnaK type molecular chaperone CSS1 were checked. In betasatellite-infected leaves, the transcript level of both the aforementioned proteins was significantly down-regulated (4- and 10-fold, respectively) (Fig. 11C). Thus, betasatellite affected both the structural and functional aspects of the host cell chloroplast by inhibiting the proteins important for chloroplast development.

The study regarding the photosynthetic efficiency revealed severe reduction in the PI of the leaf as a result of betasatellite infection. The OEC splits water molecules, and the resultant electron from the water replaces the electrons in the P680 component of PSII. The electron traverses through intermediate carriers such as pheophytin and plastoquinones, and finally ferredoxin NADP^+^ reductase (FNR) transfers one electron from each of the two ferredoxin molecules to a terminal two-electron acceptor NADPH. Transcript analysis of two major proteins in the OEC, namely PsbO (33kDa) and PsbP (23kDa), and FNR itself showed a 20-, 7-, and 3-fold decreased level of transcript accumulation in betasatellite-infected leaves, respectively (Fig. 11C). This blockage of the electron transport chain is one of the major reasons for inefficient photosynthesis. Relative amounts of mRNA transcripts were measured from leaves at different stages of infection (at 14, 21, and 28 dpi). A similar pattern of relative abundance of transcripts was observed (Supplementary Fig. S5 at *JXB* online).

## Discussion

Mosaic/chlorotic symptom formation induced by RNA viruses is a well-studied phenomenon which involves various strategic interactions with host factors (Abbink *et al.*, 2002; Lehto *et al.*, 2003). Association of a cognate or transreplicative betasatellite has been found to be ubiquitous in monopartite geminivirus-infected yellow-veined bhendi, croton, eupatorium, and malvastrum plants. Earlier observation suggested that the vein clearing symptom is not induced by DNA A of ToLCNDV alone but is caused in association with RaLCB (Singh *et al.*, 2012). In the present study, the role of βC1 in betasatellite-induced vein clearing was established by the fact that RaLCB lacking βC1 failed to induce vein clearing in plants. Interference of TYLCCNV βC1 with the AS1–AS2 protein network results in leaf deformities (Yang *et al.*, 2008). The present study deciphered the physiological and molecular details of geminivirus betasatellite-associated vein clearing symptom formation in *N. benthamiana.*

In most plant–virus systems, chlorotic symptoms lead to a decrease in the photosynthetic rate by reducing chlorophyll with a usually unchanged chlorophyll *a*/*b* ratio. However, in the case of *N. benthamiana*–RaLCB interaction, degradation of chlorophyll is associated with an ~15% increase of the chlorophyll *a*/*b* ratio, indicating that there might be preferential loss of LHCII.

Betasatellite-induced change in the chloroplast ultrastructure was associated with a large accumulation of starch inside the chloroplast, disrupted stacking of grana, reduced thylakoid content, and increased amount of plastoglobulin. Disruption of sugar loading into the phloem and perturbation of the metabolic activity of the sink leaves might lead to increased accumulation of starch in local and systemic leaves (Bechtold, 2005). Plastoglobules contain a high level of α-tocopherol which protects the thylakoid membrane, especially PSII, against photoinhibition and oxidative stress (Havaux *et al.*, 2003). Hence, accumulation of plastoglobulins might be a stress response of plants towards betasatellite-influenced damage of the thylakoid membrane and the photosynthetic apparatus.

Analysis of chlorophyll *a* fluorescence is an informative tool in understanding the effect of different environmental stresses on photosynthesis (Stirbet and Govindjee, 2011). The JIP test is based on the theory of energy flow in thylakoid membranes. Absorption (ABS) of light by PSII antenna pigments is the first step of the energy cascade which ends with PSI-driven reduction of the end electron acceptors at the PSI electron acceptor side (RE). The trapping flux (TR_0_) and the electron transport flux (ET) are the intermediate energy fluxes. In betasatellite-infected plants, the area above the chlorophyll fluorescence curve between *F*_0_ and *F*_m_ decreases, indicating a reduced plastoquinone pool size resulting in inhibition of electron transport from RCs to the plastoquinone pool (Strasser *et al.*, 2000). *F*_v_/*F*_m_, which varies very little for higher plants, with a value close to 0.834, is proportional to the photosynthetic rate (Bjorkman and Demmig, 1987). Although *F*_v_/*F*_m_ or the photochemical efficiency of PSII of dark-adapted A+β-infected plants showed a marginal reduction, the net photosynthetic rate (Pn) of A+β-infected plants decreased severely with respect to mock- and A-inoculated plants, indicating that both the dark and light reaction of photosynthesis is hampered severely with betasatellite infection (Supplementary Table S3 at *JXB* online).

The efficiency of the OEC (*F*_v_/*F*_0_), the most sensitive component of photosynthetic electron transport, is decreased in A+β-infected leaves. Damage to the OEC is indicated by a K peak in a typical OJIP chlorophyll fluorescence induction curve. The more prominent K peak in βC1-infiltrated leaves appears to be an interesting aspect of the observation (Fig. 6).

As per the leaf models, the number of active RCs and calculated energy flux for electron transport (ET/ABS) are reduced significantly in betasatellite-infected leaves. Accumulation of inactive RCs is related to the increased efficiency of dissipation of absorbed light as heat (DI_0_/RC), indicating a higher level of the non-photochemical de-excitation process. Betasatellite infection causes a 35% increase in ABS/RC, a measure of the apparent antenna size. This can be attributed either to inactivation of RCs which have been transformed to non-Qa RCs or to an increased size of the functional antenna (i.e. the antenna that supplies excitation energy to active RCs). However, the efficient absorption of photon flux by the antenna chlorophyll molecule is decreased by hampered chlorophyll biogenesis. This implies that inactivation of RCs contributes to the increased ABS/RC value.

The performance index (PI_total_) of dark-adapted intact leaves truly reflects the plant’s activity such as growth or survival under variable environmental conditions. The PI_total_ of A- and A+β-infected plants was reduced to 12% and 60% of that of mock-inoculated plants, respectively. This decrease was 40% in systemic leaves from plants transiently expressing βC1 in comparison with the mock-infiltrated plants.

Interestingly, the ultrastructural change in chloroplasts of betasatellite-infected leaves was not related to the reduction in the number of the chloroplasts. Hence, all the manifested effects are likely to be associated with the modification of the chloroplast’s intrinsic structural and functional arrangements rather than with quantitative change of chloroplasts.

The proteomic profile shows that betasatellite infection down-regulated a number of nucleus-encoded chloroplast-targeted as well as chloroplast-encoded proteins. Chloroplast Clp proteases, localized in the stroma, are structurally the most diverse Clp protease family in vascular plants. ClpP1, the only plastid-encoded Clp protease, is indispensable for chloroplast development and cell viability in tobacco (Shikanai *et al.*, 2001). Apart from Clp proteases, chloroplasts have numbers of other proteases such as FtsH and Deg which are involved in biogenesis and maintenance of PSII (Chi *et al.*, 2012). An earlier report indicated that TMV infection resulted in reduction of both transcript and protein levels of FtSH in tobacco (Seo *et al.*, 2000). In chloroplast, MATK acts as a post-transcriptional splicing factor and affects chloroplast functions, including photosynthesis (Barthet and Hilu, 2007). Down-regulation of chloroplast MATK by betasatellite can result in compromised expression of those chloroplastic genes which need MATK in their processing from premature mRNA. PsbO, a nuclear-encoded 33kDa protein, is an essential component of the OEC and is speculated to be involved in the basal defence of the plant. Increased TMV accumulation was observed in a PsbO-silenced line of tobacco (Abbink *et al.*, 2002). Notably, betasatellite infection has resulted in reduction of both PsbO protein and transcript in *N. benthamiana*. One putative protein homologous to thylakoid membrane-bound PSII protein T which is assumed to be involved in dimerization of PSII (Iwai *et al.*, 2004) was detected to be down-regulated upon betasatellite infection. Allene oxide cyclase 3 (AOC3), a chloroplast-localized enzyme which takes part in the jasmonic acid (JA) biosynthesis pathway, was also found to be down-regulated. An earlier study with TYLCCNB revealed that βC1 overexpression suppresses expression of JA-responsive genes while the transcript levels of JA biosynthetic genes were unaltered (Yang *et al.*, 2008). Interestingly, no significant difference in AOC3 transcript level between A- and A+β-infected plants was observed (unpublished data). Hence, betasatellite inhibition might act at the post-transcriptional level on some target genes including AOC3.

Jasmonic acid methyl transferase (JMT), the wound-responsive enzyme that catalyses the formation of methyl jasmonate from JA (Seo *et al.*, 2000), was found to be up-regulated following RaLCB infection. Several plant viruses including *Alfalfa mosaic virus* and TMV elicit systemic acquired resistance in plants through induction of pathogenesis-related (PR) proteins. TYLCCNB is reported to suppress the expression of PR4 protein (Yang *et al.*, 2008); however, betasatellite-infected leaves show higher accumulation of PR-1 in *N. benthamiana*. Other stress- and defence-responsive proteins including *N*-acetyltransferase, small heat shock protein, and a dehydration-responsive protein were found to be up-regulated in RaLCB-infected leaves. Pyruvate dehydrogenase, a carbon metabolic enzyme, which is known to be increased in mild drought conditions, is also up-regulated in response to betasatellite infection. The alternative oxidase (AOX)-dependent signal transduction pathway mediates virus localization and limits host tissue necrosis (Love *et al.*, 2005). In *Arabidopsis*, both avirulent and virulent *Pseudomonas syringae* pv. tomato DC 3000 induced expression of mitochondrial AOX (Simons *et al.*, 1999). The present study indicates that RaLCB infection might prime plants to induce the AOX-mediated defence response against geminiviruses..

To study whether the plants response to virus was similar at an early stage of infection even when the vein clearing was not fully manifested, the level of the selected important transcripts at earlier stages of infection such as 14 dpi and 21 dpi were checked. At earlier stages, too, betasatellite infection caused similar changes in transcript levels. Hence further study concentrated on a later stage of infection when the symptoms are fully manifested in plants.

In RaLCB–plant interactions, formation of vein clearing or mosaic-like symptoms is associated with a reduced level of chlorophyll. At the cellular level, this decrease must be related either to reduction of the biosynthesis of chlorophyll or to the enhanced degradation of chlorophyll (Balachandran *et al.*, 1997). The fact that RaLCB induced down-regulation of representative genes from the chlorophyll biosynthesis pathway suggests that impaired chlorophyll formation might contribute to the chlorosis. This result is in stark contrast to the finding of Lehto *et al.* (2003) which indicated that development of chlorosis in tobacco leaves by the Flavum strain of TMV was not caused by the transcriptional down-regulation of the chlorophyll biosynthetic pathway. On the other hand, negligible reduction of transcripts of PoA and chlorophyllase indicates that the chlorophyll degradation pathway is not the target of betasatellite infection.

The compatible reaction between *Arabidopsis* and TMV-Cg up-regulates expression of senescence-related Clp proteases (Espinoza *et al.*, 2007). Betasatellite infection induces several physiological responses such as a decrease in chlorophyll content, photosynthetic inhibition, up-regulation of defence-related genes, etc., which overlap with the senescence response of plants. In this study, down-regulation of ClpP1 at both the transcriptional and protein level indicates the existence of a complex and virus–host-specific network linking senescence and the plant’s response to biotic stress. The DnaK-type molecular chaperone CSS1 is localized in chloroplast stroma and is important for unidirectional movement of precursor polypeptide chain nucleus-encoded chloroplastic proteins through translocation pores (Ivey *et al.*, 2000; Lee *et al.*, 2009). The interaction specificity between cyanobacterial ClpP3 protease (an orthologue of the chloroplast-encoded ClpP1) and the chaperone Hsp100 is indeed important for substrate translocation (Tryggvesson *et al.*, 2012). Hence, down-regulation of one or more chloroplast protease(s) along with chloroplastic molecular chaperones would severely hamper the structural and functional integrity of the chloroplast by inhibiting productive interaction of precursor polypeptides with the translocation machinery of the chloroplast envelope.

Photosynthesis happens to be the most important reaction in living plant cells. Both structural damage of the chloroplast and inactivation of one or more enzymes involved in the energy cascade would result in inefficient electron transfer between the photosystems. Chlorotic symptoms induced by *Sunflower chlorotic mottle virus* are associated with altered redox homeostasis of the host and down-regulation of both chloroplast-encoded and chloroplast-targeted genes including PsbO and FNR (Rodriguez *et al.*, 2012). These facts reinforce the finding that PsbO, PsbP, and FNR, three important enzymes involved in efficient electron transport from PSII to NADPH via PSI, are targets of geminivirus inhibition.

For efficient localization of cellular polypeptides to the chloroplast, transit peptides are prerequisite for translocation of precursor proteins into the organelle. However, lack of consensus makes the chloroplast transit peptides among the organelles most difficult to predict. The present study reveals that RaLCB-encoded βC1 protein localizes to the nucleus as well as the chloroplast even in the absence of any predicted transit peptide. The detection of βC1 in the chloroplast of RaLCB-infected leaves by immunoelectron microscopy confirms the chloroplast localization of βC1 during pathogenesis of betasatellite.

**Fig. 12.**
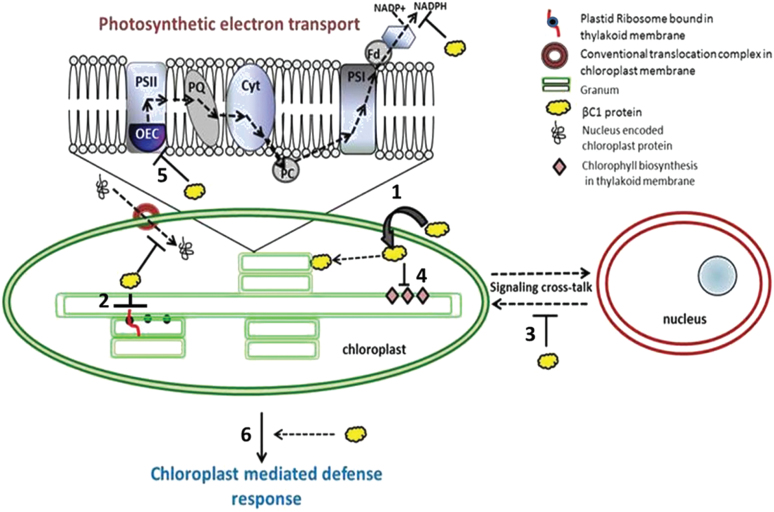
Schematic model indicating the pleiotropic role played by βC1 inside the cell affecting the chloroplast structure and function. Localization of the viral protein in the chloroplast leads to ultrastructural damage of the organelle (1). The expression of chloroplast-encoded proteins is hampered, indicating the effect of βC1 on the chloroplast translational machinery localized on the thylakoid membrane (2). Nucleus–chloroplast cross-talk is likely to be affected too as indicated (3). Biosynthesis of the chlorophyll molecule, a thylakoid membrane-bound pathway, was also curtailed (4). Exhaustive interaction of βC1 with the chloroplast led to severe photosynthetic inhibition (5), suppressed defence response (6), and subsequent symptom development. (This figure is available in colour at *JXB* online.)

To the best of the authors’ knowledge, this is the first time that the chloroplast localization of a DNA virus-encoded protein has been demonstrated. In the course of betasatellite infection, the βC1 protein localizes into chloroplasts and leads to structural deformation of the organelle and functional disruption of the photosynthetic machinery. Betasatellite infection also impedes/interferes with the chloroplast translational machinery localized on the thylakoid membrane. Thus the vein clearing symptom in plants is a consequence of βC1–chloroplast interactions as the betasatellite molecule deficient in βC1 fails to induce these changes in the plant’s physiology and plants remain asymptomatic. Taking into account all these aspects of betasatellite and host interaction, a predictive model has been proposed which would help in further exploration of the subject (Fig. 12). Further study of the molecular mechanism of chloroplast translocation of βC1 is important to explore the landscape of plant–pathogen interaction.

## Supplementary data

Supplementary data are available at *JXB* online.

Figure S1. Vein clearing symptom associated with yellow vein diseases caused by geminiviruses.

Figure S2. Graphical comparison of different parameters extracted from the chlorophyll *a* fluorescence OJIP curve.

Figure S3. Betasatellite-encoded βC1 protein localizes in the chloroplast of mesophyll cells of *N. benthamiana.*

Figure S4. Schematic diagram of the chlorophyll biosynthesis and degradation pathway.

Figure S5. Relative accumulation of host transcripts at earlier stages of accumulation.

Table S1. Transient expression of βC1 reduces chlorophyll content in systemic symptomatic leaves.

Table S2. Different biophysical parameters studied in relation to photosynthetic efficiency.

Table S3. Effect of betasatellite infection on net photosynthetic (CO_2_ exchange) rate and stomatal conductance.

Table S4. Host cellular proteins down-regulated in response to betasatellite infection.

Table S5. Host cellular proteins up-regulated in response to betasatellite infection.

Table S6. Primers used for checking the transcript level of selected host genes through qRT–PCR.

Supplementary Data
