# Supplementary material for: A geminivirus betasatellite damages the structural and functional integrity of chloroplasts leading to symptom formation and inhibition of photosynthesis
Source: J Exp Bot. 2015 Jun 25;66(19):5881–95. doi: 10.1093/jxb/erv299 (PMC4566980; doi:10.1093/jxb/erv299)
Supplement: Supplementary Data [file supp_66_19_5881__index.html]

A geminivirus betasatellite damages the structural and functional integrity of chloroplasts leading to symptom formation and inhibition of photosynthesis — A geminivirus betasatellite damages the structural and functional integrity of chloroplasts leading to symptom formation and inhibition of photosynthesis — Supplementary Data 

# A geminivirus betasatellite damages the structural and functional integrity of chloroplasts leading to symptom formation and inhibition of photosynthesis

## Supplementary Data

Data files

- Supplementary Data - Supplementary Data
