# Supplementary material for: A geminivirus betasatellite damages the structural and functional integrity of chloroplasts leading to symptom formation and inhibition of photosynthesis
Source: J Exp Bot. 2015 Jun 25;66(19):5881–95. doi: 10.1093/jxb/erv299 (PMC4566980; doi:10.1093/jxb/erv299)
Supplement: Supplementary Data [file supp_erv299_jexbot146969_file001.pdf]

SUPPLEMENTARY MATERIALS

A geminivirus betasatellite damages structural and functional integrity of chloroplasts leading to symptom formation and inhibition of photosynthesis

Dhriti Bhattacharyya, Prabu Gnanasekaran, R Kishore Kumar, Nirbhay Kumar Kushwaha, Veerendra Kumar Sharma, Mohd Aslam Yusuf and Supriya Chakraborty*

Molecular Virology Laboratory, School of Life Sciences, Jawaharlal Nehru University, New Delhi – 110 067, India.

*Author for correspondence:

Supriya Chakraborty
Professor
Molecular Virology Laboratory
School of Life Sciences
Jawaharlal Nehru University
New Delhi – 110 067
India
Tel: 91-11-2670 4153
Fax: 91-11- 2674 2558

e-mail : supriyachakrasls@yahoo.com

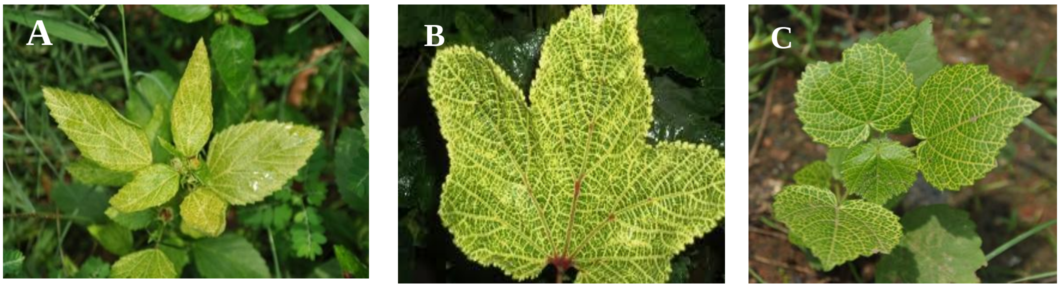

Fig. S1. Vein clearing symptom associated with yellow vein diseases caused by geminiviruses. (A) Ageratum yellow vein disease, (B) Bhendi yellow vein disease, (C) Mulvastrum yellow vein disease. Presence of betasatellites have been ascertained with each of these diseases.

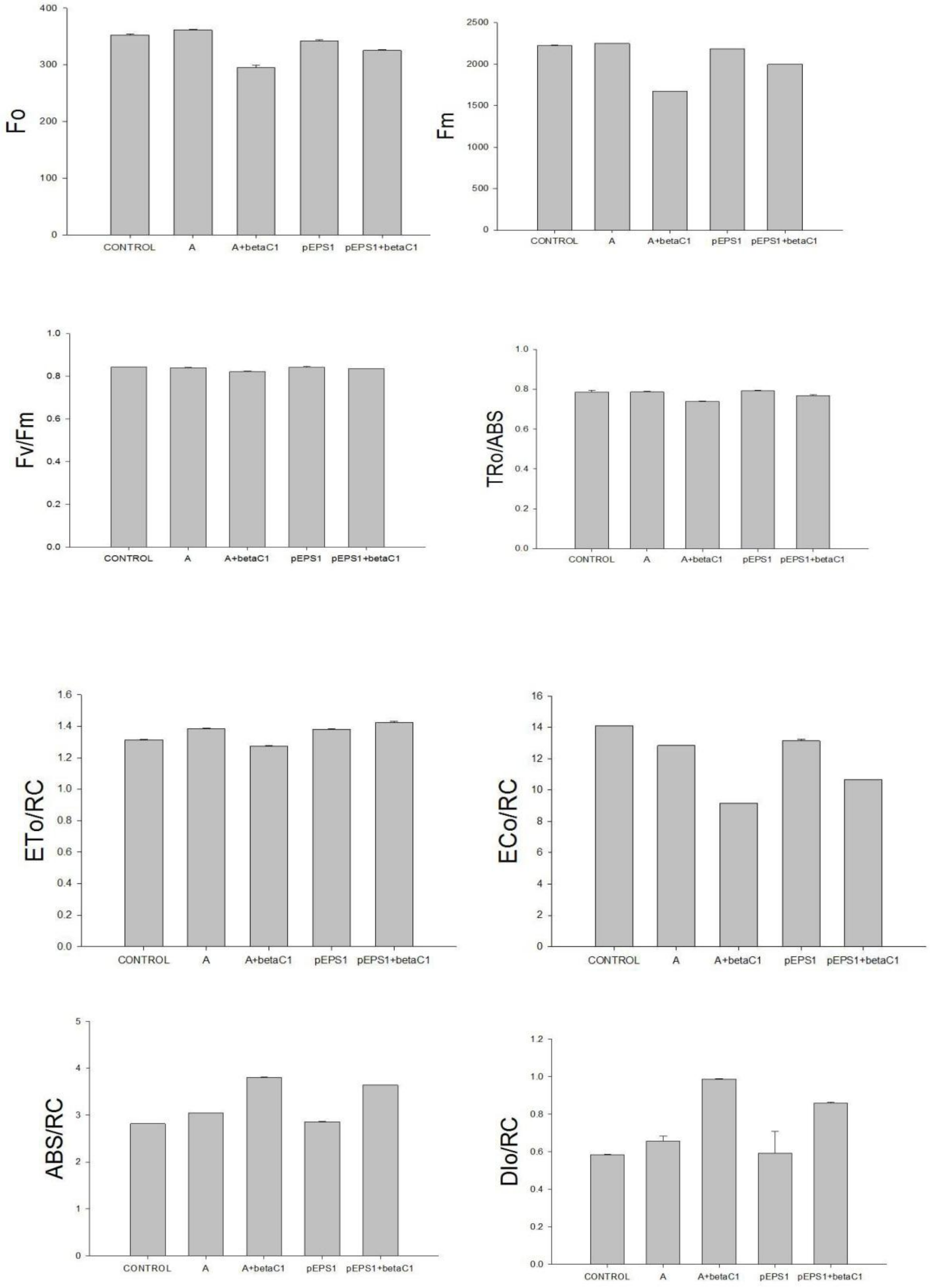

Fig. S2. Graphical comparison of different parameters extracted from chlorophyll a fluorescence OJIP curve

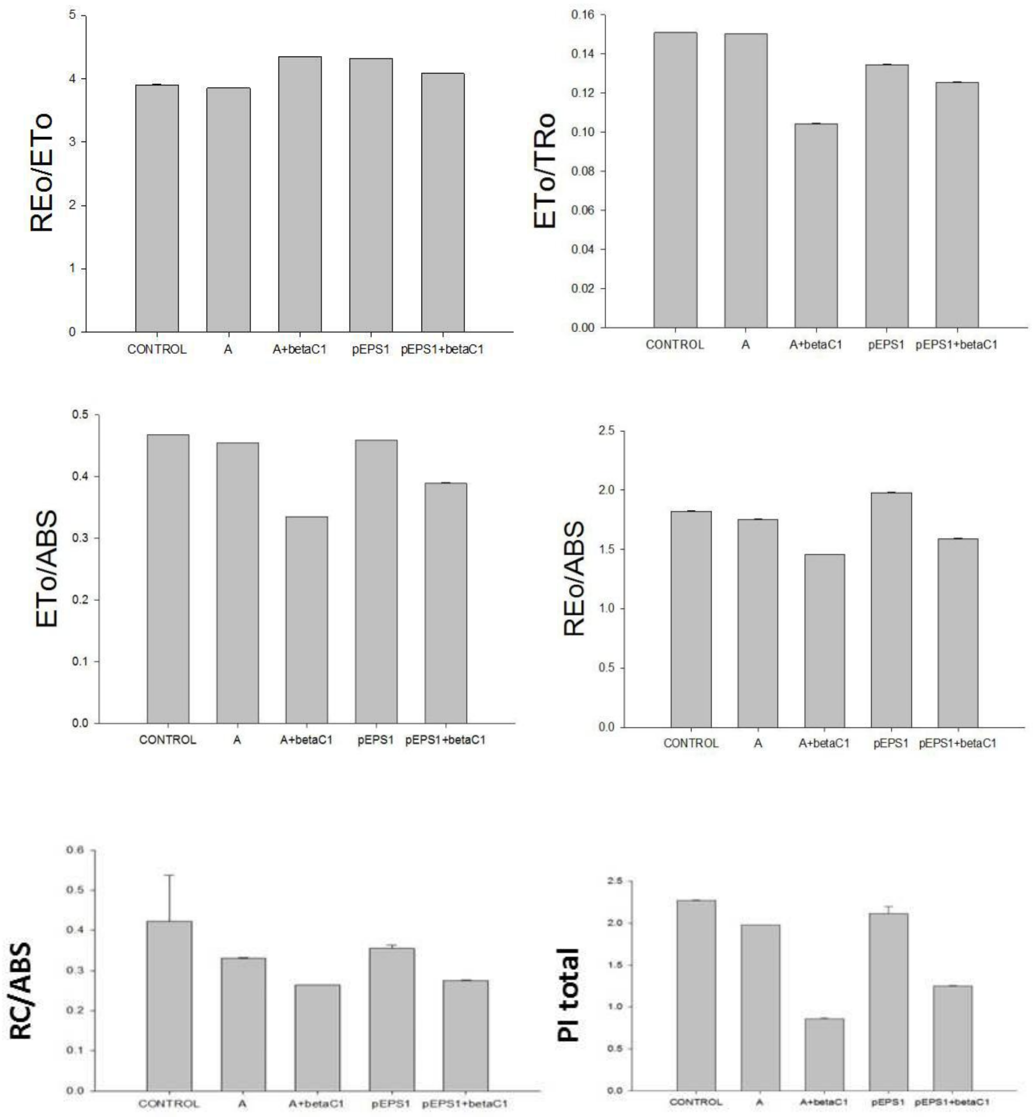

Fig. S2. Graphical comparison of different parameters extracted from chlorophyll a fluorescence OJIP curve

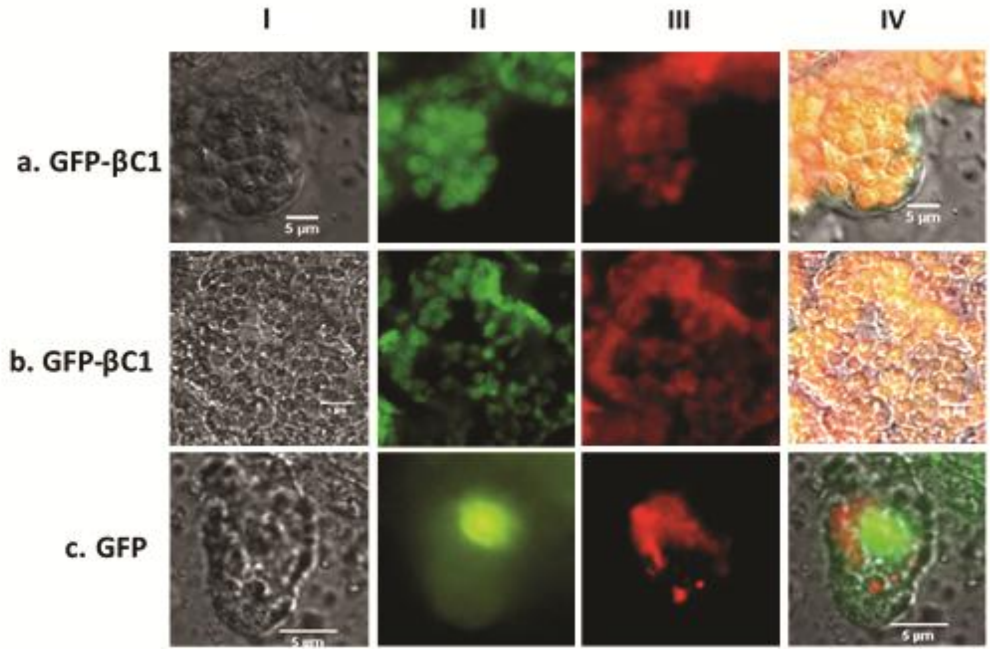

Fig. S3. Betasatellite encoded βC1 protein localizes in chloroplast of mesophyll cells of *N. benthamiana.*

Sections of *N. benthamiana* leaves, agroinfiltrated with pBIC- βC1 (row a and b) and pBIC vector (row c), were visualized under fluorescent microscope. Images of the cells (panel I) in bright field, (panel II) showing GFP fluorescence and (panel III) showing chlorophyll autofluorescence. Panel IV represents merged images of panel I, II and III.

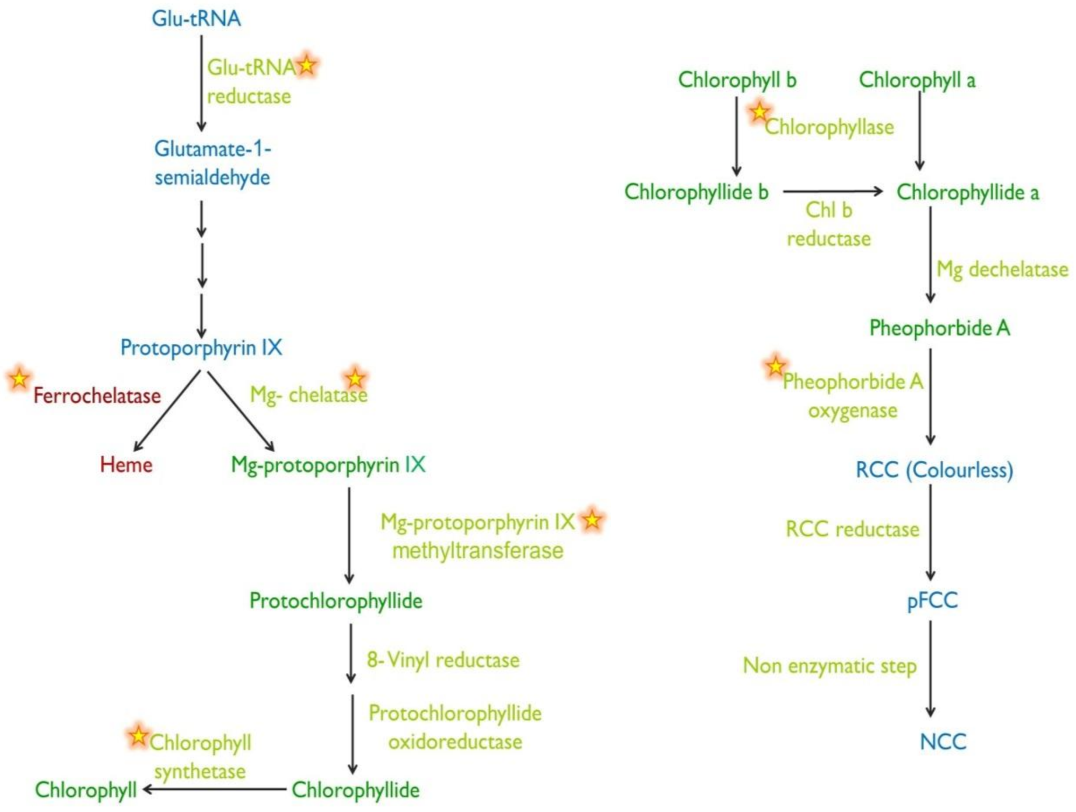

Fig. S4. Schematic diagram of chlorophyll biosynthesis and degradation pathway. Genes selected for checking the transcript level are marked asterisk.

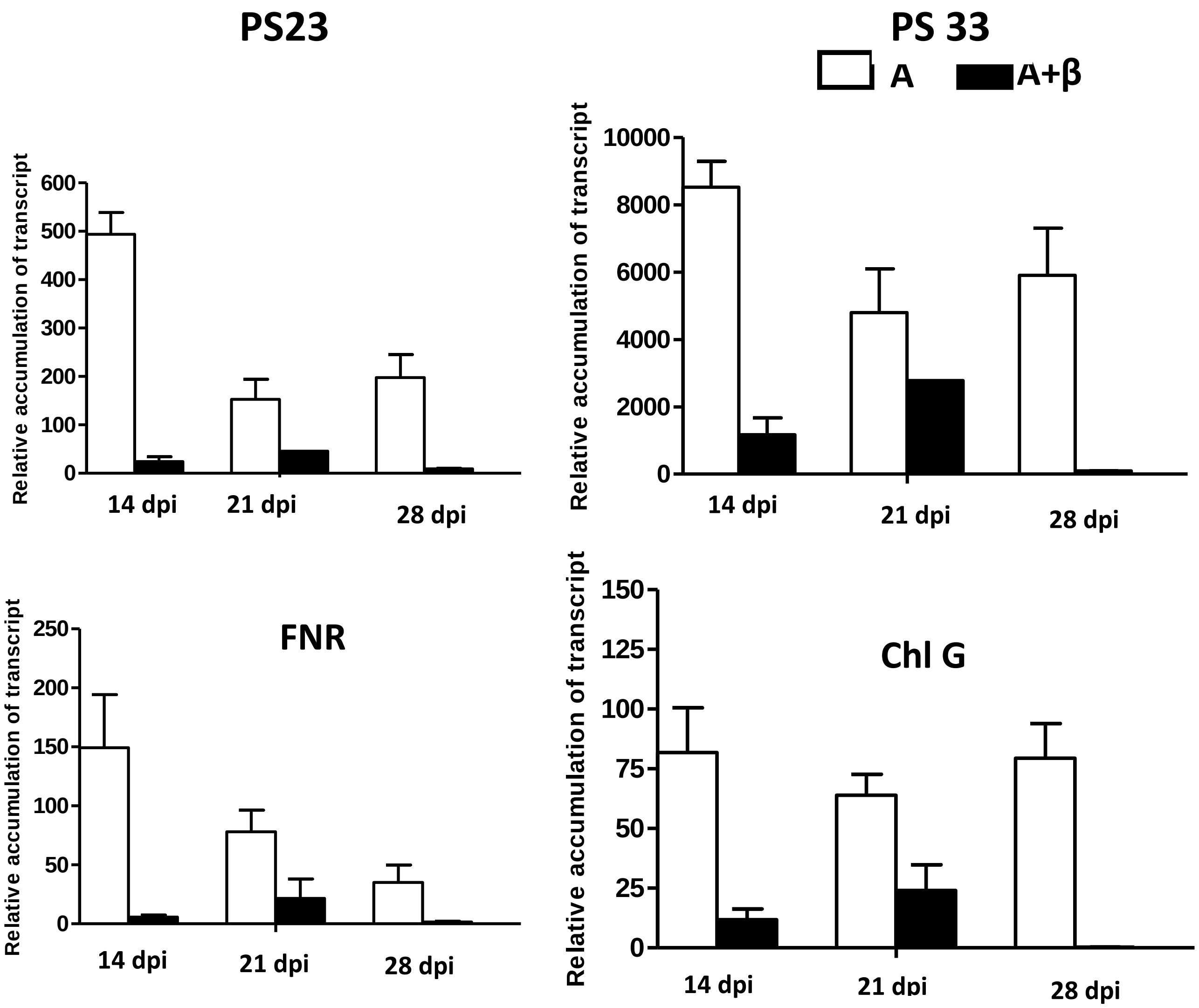

Fig. S5. Relative accumulation of host transcripts at earlier stages of accumulation

Table S1. Transient expression of βC1 reduces chlorophyll content in systemic symptomatic leaves

Construct	Chlorophyll a mg / g tissue (average)	Chlorophyll b mg / g tissue (average)	Total Chlorophyll mg / g tissue (average)
pEPS1	1.15	1.256	2.4
pEPS1+βC1	0.96	0.485	1.445

Table S2. Different biophysical parameters studied in relation to photosynthetic efficiency

Parameters to analyse Chl a fluorescence	Significance
PI ABS	Performance index (potential) for energy conservation from exciton to the reduction of intersystem electron acceptors
PI total	Performance index (potential) for energy conservation from exciton to the reduction of PSI end acceptors
ABS/RC	Absorption flux (of antenna Chls) per RC
Fv/Fm	Maximum yield of primary photochemistry or maximum quantum efficiency of Photosystem II.
TRo/CSm = Fm-Fo/CSm	Initial (maximum) flux of photochemically conserved (trapped) excitation energy per excited cross section
TRo/ABS=	Trapping flux (leading to Q_A reduction) per absorption
Eto/CSm = Fm-Fj/CSm	Flux of electrons transferred between two photosystems per excited cross section.
RE_0/ ET_o,	Efficiency/probability with which an electron from the intersystem electron carriers moves to reduce end electron acceptors at the PSI acceptor side (RE)
RE_o/ABS	Quantum yield for reduction of end electron acceptors at the PSI acceptor side (RE)
ET_o/ TR_o	Efficiency/probability for electron transport (ET), i.e. efficiency/probability that an electron moves further than Q_A^-
ET_o/ABS	Quantum yield for electron transport (ET)
ET_0/RC	Electron transport flux (further than Q_A^-) per RC
DIo/CSm =Fo/CSm	Measure of the energy dissipitatedas heat or flourescence per excited cross section.
DI_0/RC	dissipated energy flux per active reaction centre
RC/ABS	Q_A-reducing RCs per PSII antenna Chl (reciprocal of ABS/RC)
EC_0/RC	Electron transport flux (further than Q_A^-) per RC

Table S3. Effect of betasatellite infection of on net photosynthetic (CO_2exchange) rate, and stomatal conductance

Treatment	Photosynthetic efficiency % (w.r.t control)	Stomatal conductance % (w.r.t control)
A inoculated (28 dpi)	96	84
A+β inoculated (28 dpi)	38	85
pEPS1 vector infiltrated (7dpi)	94	62
pEPS1+βC1 infiltrated (7 dpi)	69	75

Table S4. Host cellular proteins downregulated in response to betasatellite infection

Spot no.	Protein homology	Acc. No.	Theoretical/Experimental		% Change[a]	Coding site/ Localization
			Mass	pI		
A01-05	Rubisco LSU	ABC86677	49.88/50.45	6.8/6.79	45±2.18	chloroplast /chloroplast
A06-08	Rubisco SSU	AAA34111	10.3/11.3	5.3/5.5	40±4.06	nucleus /chloroplast
A09	ATP-dependent ClpP1 protease	ABF95892.1	37.16/37.1	9.45/9.9	33±2.14	nucleus /chloroplast
A10	Phosphoglycerate kinase	NP_001147628	50.01/50	6.07/6.1	35±0.3	nucleus /cytoplasm
A11	NBS-coding resistance gene analog	ACE79518.1	30.89/30.9	5.4/5.6	30± 4.97	nucleus /cytoplasm
A12	Leucine zipper protein-like	gi|56784542	44.6/43.2	6.84/7	27±2.10	nucleus /cytoplasm
A13	Maturase K	NP_566734	35.94/35.9	9.74/9.9	25±7.18	nucleus /chloroplast
A14	Ribosome production factor 2-like protein	NP_566734	34.4/37.5	9.1/8.7	20±1.27	nucleus /cytoplasm
A15	Photosystem II protein T	ABQ15043	40.1/40.03	9.52/10.0	47±7.47	nucleus /Chloroplast membrane
A16	33kDa protein of Oxygen evolving complex	Q157M6	21.9/21.2	5.8/5.92	33±1.45	nucleus /Chloroplast membrane
A17	Allene oxide cyclase 3, chloroplast precursor	XP_002524412	28.71/21.7	9.32/9.7	25±1.24	nucleus /Chloroplast membrane
A18	Glutamate decarboxylase	XP_003616074	17.4/17.9	10.32/10.4	30±3.8	nucleus /cytoplasm

[a]Proteins were considered as differentially expressed when a 1.2-fold increased or decreased accumulation was statistically confirmed by one-way ANOVA with a *p* value 0.05. The fold change was calculated comparing each sample versus the corresponding control. Differentially expressed proteins were identified by MALDI TOF/TOF.

Table S5. Host cellular proteins upregulated in response to betasatellite infection

Spot no.	Protein homology	Acc. No.	Theoretical/Experimental		% change[a]	Coding site/ Localization
			Mass	pI		
B01	Pathogenesis-related protein 1-1a	gi|253796028	14.24/14.8	6.92/7.2	15±1.48	nucleus /cytoplasm
B02	N-acetyltransferase	XP_003597800	37.9/35.5	9.21/9.01	21±1.03	nucleus /cytoplasm
B03	Mitochondrial alternative oxidase 1b, partial	ACI28870	23.2/25.6	6.22/6.23	23±1.63	nucleus /mitochondria
B04	Fructokinase	NP_564875	4.173/ 5.2	5.54/5.5	22±3.0	nucleus /mitochondria
B05	Small heat-shock protein	CAC81964	18160	5.7/5.9	21±3.18	nucleus /cytoplasm
B06	Early responsive to dehydration	gi|319993043	15.3/15.34	4.53/4.4	24±3.5	nucleus /cytoplasm
B07	Jasmonic acid methyl transferase	AT1G19640	37.9/35.5	10.32/10.4	34±2.8	nucleus /cytoplasm
B08	Heat shock protein 17.0	CAA63901	16.95/16.5	5.78/5.6	20±.1.8	nucleus /cytoplasm
B09	Pyruvate dehydrogenase	NP_174703	48.3/45.6	8.8/8.2	23±1.05	nucleus /cytoplasm

[a]Proteins were considered as differentially expressed when a 1.2-fold increased or decreased accumulation was statistically confirmed by one-way ANOVA with a *p* value 0.05. The fold change was calculated comparing each sample versus the corresponding control. Differentially expressed proteins were identified by MALDI TOF/TOF.

Table S6. Primers used for checking transcript level of selected host genes through qRT-PCR

Target transcript	Forward primer	Reverse primer	Amplicon size
Magnesium (Mg) protoporphyrin IX chelatase subunit I (CHLI)	NtRTCHLIFP 5'GGAAATCAACCTGCTC AAGAACA 3'	NtRTCHLIRP 5'ATCATCACACCTCC AATCTTTGG 3'	150bp
Mg protoporphyrin IX chelatase (CHLH)	NtRTCHLHFP 5'CCCTTTGGCTCCTTGTA TGTATG 3'	NtRTCHLHRP 5'GCCCAACAACAGG AGCATT3'	110bp
Mg protoporphyrin IX chelatase (CHLD)	NtRTCHLDFP 5'GGCCATTGACCGTGAG ATAGG3'	NtRTCHLDRP 5'CATTTGCCATTGAG CCAACA3'	128bp
HEMA1 encoding glutamyltRNAreductase	NtRTHEMAFP 5'GGGTCGGGTCAGACGA TTG3'	NtRTHEMARP 5'CAAGAGCAGAAAG GCTGGAAGA3'	137bp
Ferrochelatase	NtRTFeChFP 5'GAGAAAAGCACTGCGG GAAAG3'	NtRTFeChRP 5'GAGGATAAAGAGG GAGCACAACA 3'	140bp
Mg-protoporphyrin IX methyltranserase (Chl M)	NtRTChlMFP 5'AGTCCCCGGCACCAGT AATG 3'	NtRTChlMFP 5'CAGCCAATGACGCA AGATGA 3'	150bp
Chlorophyll synthase (CHLG)	NtRTChlGFP 5'CCCTTTTGCCTCTCATT TTCC 3'	NtRTChlGRP 5'TCCACTTGTCCGTTT CTTGCT 3'	163 bp
Chlorophyllase	NtRTPhyllaseFP 5'CCCCATCAGAAGCAGG AAAC 3'	NtRTPhyllaseRP 5'TGCTCCTTCCACCA AATATAACTG 3'	146 bp
Pheophorbide A oxygenase 1	NtRTPheoFP 5'TTCAGCTCCTCAATCGA GACTTAGT 3'	NtRTPheoRP 5'GATAAAGGAGCAA GGCGATGA 3'	100bp
PS23 OEC PSII	NbRTPS23FP 5'CAATGCTTCTTGCACCA TCAT 3'	NbRTPS23RP 5'TTGAGGTGCAGACT GTTTTTGG 3'	126BP
PS33 OEC PSII	NbRTPS33FP 5'CCCACATCATTCACGGT CAA 3'	NbRTPS33RP 5'CGAATGGTCCCTCA ATCTCA 3'	118BP
Clp Protease	NtRTClpFP 5'GCGGAAGAACTGCTGA AACTG 3'	NtRTClpRP 5'TTGGGCTTCTGTTG CTGACA 3'	110 BP
Ferredoxin-NADP reductase	NtRTFdnrFP 5'GCTCCAAAAAGACAAC ACCTTCA 3'	NtRTFdnrRP 5'CCGCCCAGACAATA CCATCT 3'	110BP
Dna K type molecular chaperone CSS1precursor chloroplast	PsRTDnaKFP 5'TGATGGGCAGACAAGT GTAGAAA 3'	PsRTDnaKRP 5'GAGGAGCGGGAGG GATACC 3'	110bp
